# Artificial Intelligence in Thoracic Surgery: Transforming Diagnostics, Treatment, and Patient Outcomes

**DOI:** 10.3390/diagnostics15141734

**Published:** 2025-07-08

**Authors:** Sara Lopes, Miguel Mascarenhas, João Fonseca, Maria Gabriela O. Fernandes, Adelino F. Leite-Moreira

**Affiliations:** 1 Portuguese Institute of Oncology of Porto, 4200-072 Porto, Portugal; 2 Faculty of Medicine, University of Porto, 4200-437 Porto, Portugal; 3 Precision Medicine Unit, Department of Gastroenterology, Hospital São João, 4200-437 Porto, Portugal; 4 WGO Training Center, 4200-437 Porto, Portugal; 5 Institute for Research and Innovation in Health—Associate Laboratory (i3s-LA) (IPATIMUP/i3s), 4200-135 Porto, Portugal; 6 Department of Cardiothoracic Surgery, Hospital São João, 4200-437 Porto, Portugal

**Keywords:** artificial intelligence, bioethics, big data, thoracic surgery, deep learning, machine learning, natural language processing, SaMD, data protection

## Abstract

**Background/Objectives:** Artificial intelligence is revolutionizing healthcare. In the recent years, AI tools have been incorporated by medical specialties that heavily rely on imaging techniques to aid in the diagnosis, management, and monitoring of a wide array of clinical conditions. **Methods:** Thoracic surgery is not an exception: AI is becoming a reality, although it is only the beginning. AI-based tools can be employed in medicine, and by extracting useful information from big data, they allow for the early diagnosis of diseases like lung cancer. Diagnostic imaging is the most promising clinical application of AI in medicine. **Results:** As for other specialties, ethical issues represent a challenge in thoracic surgery and must be addressed before introducing these applications. Data protection and biases, privacy, ‘the black box’ problem (explainability), and responsibility are some challenges that AI must supplant. **Conclusions:** In this review, the authors aim to highlight the importance of AI in thoracic surgery. AI applications, future directions, and clinical benefits and challenges, particularly in this area, will be addressed, highlighting solutions to successfully incorporate AI into healthcare protocols.

## 1. Introduction

Artificial intelligence (AI) was born within the Dartmouth Summer Research Project (1956, Hanover, Grafton County, New Hampshire (USA). [1]. Almost 50 years ago, an intensive two-month project tried to obtain solutions to the problems faced when building a machine that simulates human intelligence [1,2,3,4,5,6].

In recent years, AI has been actively implemented in healthcare, changing medicine with solutions in diagnostics, treatment planning, and personalized medicine, thus helping in clinical decision making. The era of digitalized medical imaging is allowing for advances in AI, including the design of AI solutions to aid image acquisition and analysis, on which several medical specialties rely to determine disease diagnosis and prognosis, monitor disease progression, and in more recent times, even deliver therapeutic interventions [5,7,8,9,10,11,12,13,14,15,16]. Gastroenterology is one of the areas where AI has been the most applied: The application of capsule endoscopy (CE) with AI has been revolutionary, allowing for the detection of certain features in the obtained images [2,9,17,18,19].

AI is expected to revolutionize the diagnosis of lung disease, the early detection of lung cancer (LC), surgical precision, and personalized treatment. Studies show an important role for AI in LC imaging analysis, optimizing the precision of screening and the efficiency of clinicians [2,20,21,22,23,24,25,26,27]. Patient outcomes can also be improved by optimizing thoracic surgeries, including video-assisted (VATS) and robotic-assisted (RATS) thoracic surgery [23,28,29].

The use of AI in medicine is increasing exponentially with AI tools breaking new ground but respecting specific criteria to ensure that they are trustworthy [30,31]. Achieving the vast opportunities that AI can offer in medicine, it is necessary to address ethical and safety issues [32,33]. The authors aim to review the current state of the art of AI in TS, discussing capabilities, implications, challenges, and future applications.

### Overview of Artificial Intelligence in Medicine: Definitions, Types, and Subtypes

**Figure 1:**
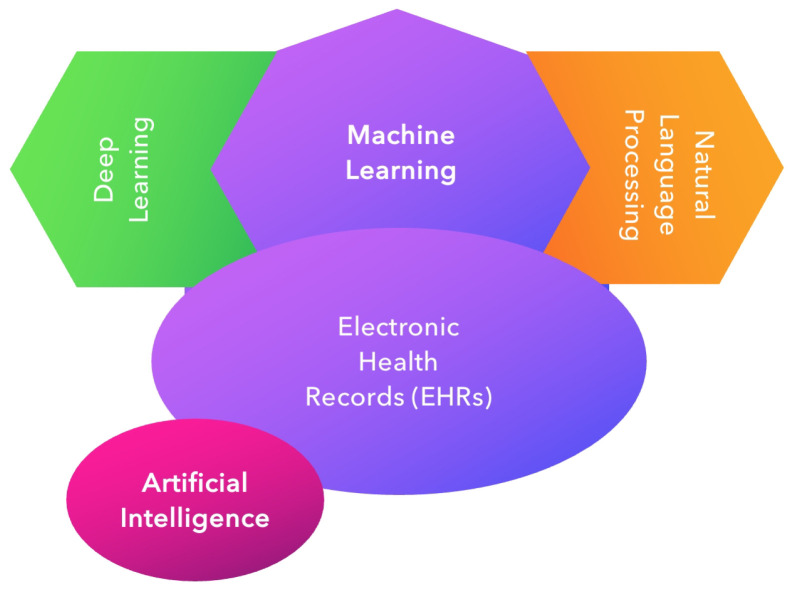
Computerized algorithms of AI to analyze EHRs, identifying complex data beyond human capacity.

AI simulates, extends, and expands human intelligence. It covers tasks such as reasoning, learning, language processing, and the display of knowledge or information [32,34,35,36]. This is the era of big data: the digitalization of clinical data and the widespread use of electronic health records (EHRs) [37]. AI uses computerized algorithms to dissect these complicated data, encompassing various techniques, such as machine learning (ML), deep learning (DL), and natural language processing (NLP), showing some overlapping features [2,22,35,38,39,40,41]. These methodologies allow AI systems to analyze large volumes of medical data, identifying complex patterns [42,43,44] (Figure 1).

**Figure 2:**
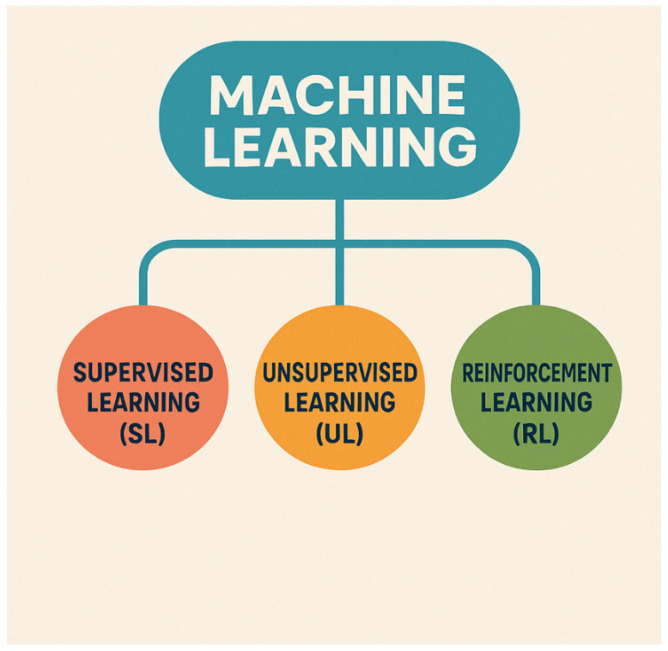
Subtypes of machine learning.

Table 1 explains the different subtypes of AI algorithms [30,45,46,47,48,49,50,51]. Useful information from unstructured data (clinical reports, operative notes, and discharge summaries) can be obtained by NLP technology. Narrative texts are transformed into data that computer programs can process [22,34,41]. ML tools construct analytical algorithms to iteratively analyze a huge amount of structured information, such as imaging and genetic data, and are able to extract meaningful patterns, creating prediction models about input variables [38,44,52,53]. ML is divided into three subtypes: Supervised Learning (SL), Unsupervised Learning (UL), and Reinforcement Learning (RL) [7,8] (Figure 2). SL is about training a model from input variables and their corresponding labels, using a dataset labeled by humans; SL can recognize different features independently, providing an output of the predictions in a large quantity of information [54]. Conversely, UL includes algorithms where the only inputs are raw features (outcomes are unknown), related to training a model to find patterns in an unlabeled dataset; UL can find hidden patterns in the data without human input [38,44,52].

**Figure 3:**
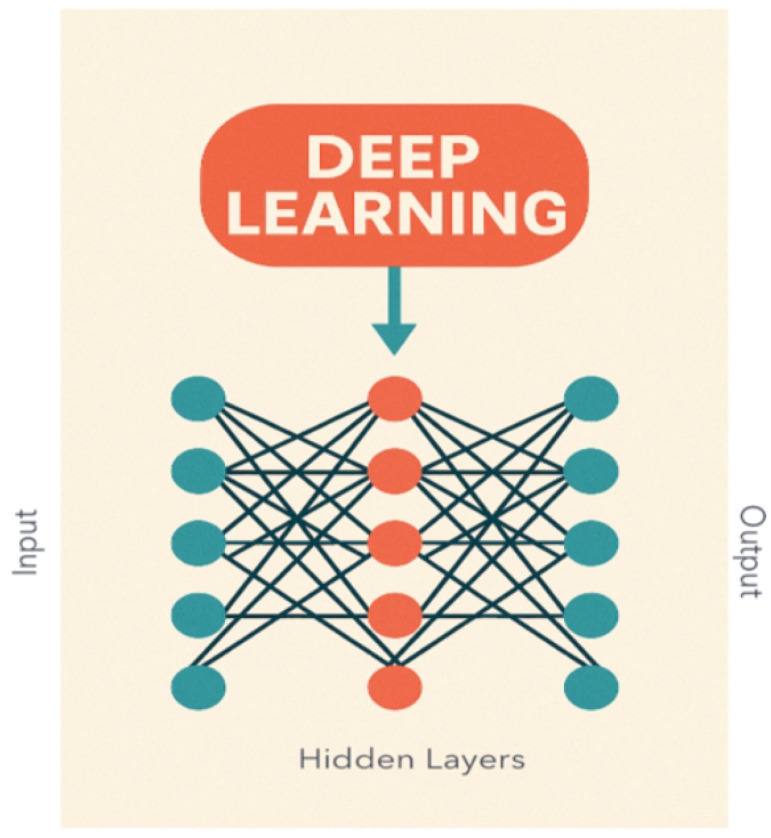
DL is an ML subtype which creates a relation between input variables and outcomes of interest.

RL is a type of ML where an agent (inspired by behavioral psychology) takes actions (what the agent can do) in the environment (where the agent operates), receives rewards (positive feedback) or penalties based on those actions, and can learn to maximize total rewards over time. RL algorithms are the core technique at the heart of robotic surgery: RL is already proving valuable in supporting, improving, and personalizing surgical robotics [34,44,45,55,56,57]. DL, a subtype of ML, handles complex neural networks (organized in multiple layers, which allows them to perform complex tasks), based on the biological functioning of the human brain, creating a relation between the input variables and the outcomes of interest (Figure 3). DL algorithms include Convolutional Neural Networks (CNNs), deep neural networks (DNNs), recurrent neural networks (RNNs), and deep belief networks (DBNs) [39,58]. DNNs use a hierarchy of signals in which higher-level features are obtained by combining lower-level ones. CNNs, a subtype of DNN, resemble neurobiological processes, being responsible for image analysis, through the connectivity pattern between neurons (abnormality detection, disease classification, and computer-aided diagnosis). CNNs have superior performance in object detection and recognition, being less dependent on human effort [20,21].

**Figure 4:**
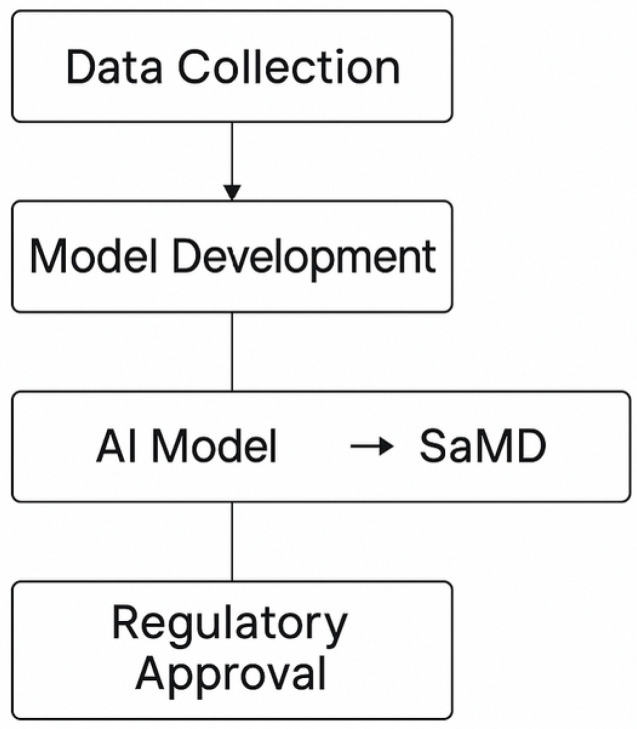
Exemplification of how an AI model becomes part of SaMD.

**Figure 5:**
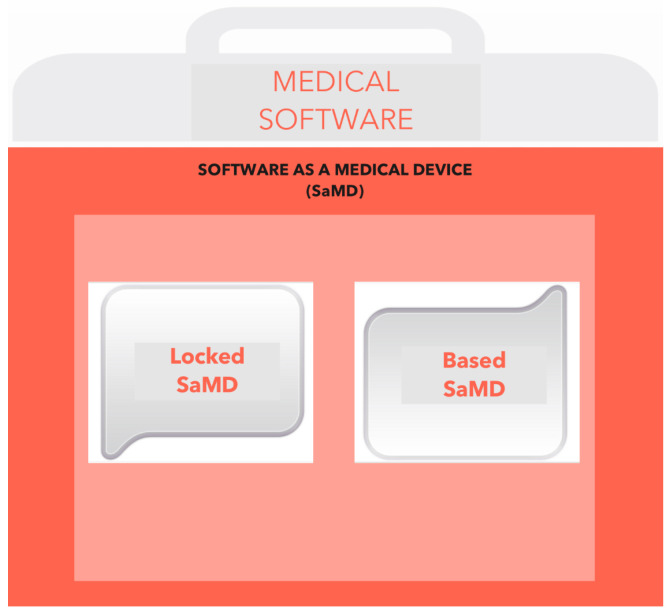
Subtypes of Standalone Software as a Medical Device (SaMD).

The digitalization of healthcare introduced informatic solutions for data management allowing for the use of semi- and fully automated analytical software tools that can be integrated into Standalone Software as a Medical Device (SaMD) [20,58,59,60,61,62] (Figure 4). Thoracic surgery (TS) is an area highly reliant on imaging, highlighting the exponential growth of SaMD in healthcare [61,62]. There are locked SaMD tools and AI-based SaMD tools. Locked SaMD applications are static devices that produce the same result when given the same input. AI-based SaMD applications are dynamic and capable of learning and adapting over time, potentially improving performance. This technology can also leverage big data in conjunction with information from EHRs, generating an unparalleled set of efficient resources (Figure 5). The future of AI in diagnostic procedures will undoubtedly involve ancillary SaMD [61,62].

Gastroenterology presents clear evidence that SaMD applications are powerful tools for healthcare professionals and patients (e.g., AI-assisted endoscopy and virtual colonoscopy software) [12,13,17,18,31,63]. SaMD can be incorporated into endoscopy apparatus, such as CE; the incorporation of ML algorithms provides real-time image analysis, standardizing diagnostic accuracy with no dependence on the endoscopist’s experience [26,64]. ML initially involves setting up an algorithm (including data preparation, feature engineering, and relevant features) to evaluate important image elements. The algorithm then identifies the combination of features that best classify the image or defines a metric for a specific image region. Furthermore, DL approaches have been employed to develop scoring systems that help stratify risk and predict prognosis or response to treatment, for example, by predicting patient survival or identifying patients that might benefit from biological therapies [2,21,31,61,65]. Table 2 shows the difference between AI algorithms and SaMD. SaMD-based telemedicine platforms facilitate remote consultations, improving patient access to care, especially for those who are far from specialized settings or have limited mobility. In addition, the use of SaMD to manage administrative tasks and data handling at healthcare facilities can also improve clinical outcomes and reduce costs [62].

AI application goals underscore the larger mission of AI in medicine: transform healthcare delivery, improve diagnostic and treatment precision, and improve patient outcomes while reducing costs and administrative burdens [8,30]. It is important to understand how the benefits will impact different healthcare systems around the world.

## 2. AI in Diagnosis and Disease Detection

### 2.1. Medical Imaging

By integrating medical imaging EHRs and real-time patient data, AI has played a transformative role in early detection, disease monitoring, management, and prediction of respiratory infections (COVID-19, pneumonia, tuberculosis, influenzae, etc.) and LC [37,43,66,67,68,69,70]. AI tools must seamlessly integrate with PACSs (Picture Archiving and Communication Systems) for integration with radiology workflows [8,31,33].

**Figure 6:**
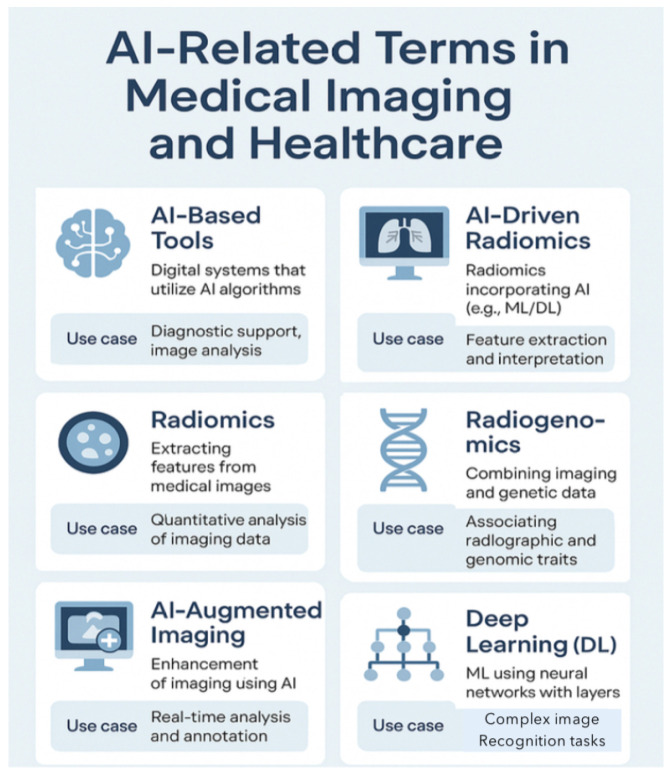
AI-related terms in medical imaging and healthcare.

AI potentiates the interpretation of lung ultrasound (LUS), chest X-rays (CXRs), computed tomography (CT), and magnetic resonance imaging (MRI) [14,20,52,71,72,73]. AI has shown impressive precision and sensitivity in the identification of imaging abnormalities, both for diagnosis and monitoring (tissue-based detection and characterization)—changes in the imaging pattern that are not easily amenable to human detection and subtle changes of indeterminate significance [2,17,18,20,31,52,58,71,72]. Table 3 and Figure 6 explain common AI medical terms in medical imaging [17,22,30].

#### 2.1.1. CXR, CT Scan, and LUS: Chronic and Infectious Lung Diseases

AI is improving CXR interpretation, particularly in detecting lung diseases (Table 4) [21,74,75,76,77,78]. Several studies, including those by Rajpurkar et al. [79], have demonstrated that AI tools can analyze CXR images to detect abnormalities such as lung nodules, infiltrates, and pleural effusions; classify diseases (e.g., viral vs. bacterial pneumonia); quantify the severity of conditions like interstitial lung disease (ILD); and support triage by prioritizing urgent cases for radiologists (AI-assisted triage) [21,39,59,60,66,80,81,82,83,84,85,86,87]. In emergency settings, AI-assisted CXR analysis enhances early diagnosis and intervention in conditions like pulmonary edema (PE) and heart failure (HF), as demonstrated by Annarumma et al. Oxipit, a health-tech company, has developed an AI algorithm called ChestEye, a web-based automatic computer-aided diagnosis system capable of detecting 75 pathologies, covering approximately 90% of potential radiological findings [88].

**Table 4:** Studies of AI in CXR and CT scans in pulmonology and thoracic surgery.

Author/ Paper	Year of Publication	Field of Application	Dataset	AI Type	Results
Eppenhof et al. [74]	2017	To develop a deformable registration method based on a 3D convolutional neural network, together with a framework for training such a network	The network directly learns transformations between pairs of 3D images, and is trained on synthetic random transformations which are applied to a small set of representative images for the desired application	CNNs	Results: an accurate and very fast deformable registration method Without requirement for parametrization at test time or manually annotated data for training
Nibali et al. [75]	2017	To improve the ability of CAD systems to predict the malignancy of nodules from cropped CT images of lung nodules	Directly compare the system against 2 state-of-the-art DL systems for nodule classification on the LIDC/IDRI dataset using the same experimental setup and dataset. Using the state-of-the-art ResNet architecture as basis	Deep residual networks (ResNets)	- The system achieves the highest performance in terms of all metrics measured including sensitivity, specificity, precision, AUROC, and accuracy
da Silva G et al. [76]	2018	Propose a methodology to reduce the number of false positives using a DL technique in conjunction with an evolutionary technique The methodology was tested on CT scans	- The PSO algorithm was used to optimize the network hyperparameters in CNN, in order to enhance the network performance and eliminate the requirement of manual search	CNNs	Lung nodule false positive reduction on CT images Accuracy = 97.62% Sensitivity = 92.20% Specificity = 98.64% AUROC curve of 0.955
Choi et al. [77]	2018	To develop a radiomics prediction model to improve pulmonary nodule classification in low-dose CT To compare the model with the Lung-RAD Early detection of LC	- A set of 72 PNs (31 benign and 41 malignant) from the LIDC-IDRI - 103 CT radiomic features were extracted from each PN	Support vector machine and LASSO	- Accuracy = 84.1% (11.9% higher than Lung-RADS) - AUC = 0.71–0.83 (indicating strong discriminative ability)
Nam et al. [21]	2019	To develop and validate a DLAD for malignant PN on chest radiographs To compare its performance with physicians including thoracic radiologists	43292 chest radiographs (normal radiograph-to-nodule radiograph ratio, 34,067:9225) in 34,676 patients	DL-based automatic detection algorithm (DLAD)	- Radiograph classification performances of DLAD were a range of 0.92–0.99 (AUROC) and 0.831–0.924 (JAFROC FOM), respectively - Enhanced detection of malignant PN
Bashir et al. [78]	2019	To compare the performance of random forest algorithms utilizing CT radiomics and/or semantic features in classifying NSCLC	2 thoracic radiologists scored 11 semantic features on CT scans of 106 patients with NSCLC (specifically for distinguishing adenocarcinoma and schamous cell carcinoma Total of 115 radiomic featuresextracted from CT scans	Random forest	Non-invasive classification of NSCLC can be done accurately Superior performance of models based on semantic features

AUC—area under curve; AUROC—area under the receiver operating characteristic; CAD systems -AI-derived computer-aided detection; CT scans- computerized tomography scans; DL—Deep Learning; DLAD—DL-based automatic detection algorithm; LASSO—Least absolute shrinkage and selection operator techniques; LC—Lung Cancer; LIDC-IDRI—Lung Image Database Consortium image collection; PN—Pulmonary Nodules; PSO— particle swarm optimization; CNN—the convolutional neural network; NSCLC—Non-small cell lung cancer.

CheXNet and qXR by Qure.ai have been trained on millions of CXR to identify pneumonia with precision at the radiologist level [21,23,59,74]. CheXNet, an AI-based image recognition system, developed by Rajpurkar et al. [79], outperformed radiologists in diagnosing pneumonia by analyzing millions of labeled X-ray images [59,79]. AI models such as qXR by the Indian company Qure.ai were deployed in low-resource settings for rapid pneumonia screening [74,80,84,85]. AI-powered DL models, such as COVID-Net, proposed by Wang and Wong, analyze CXR and CT scans to distinguish COVID-19 from other pneumonias [66,89]. Researchers at Charles Darwin University have developed an AI model capable of diagnosing pneumonia, COVID-19, and other lung diseases from LUS videos with an accuracy of 96.57% [90,91,92,93,94]. This model analyzes video frames to identify specific lung features and patterns, allowing radiologists to make fast and accurate diagnoses (Table 5) [72,81,90,92,93,95,96,97,98,99,100].

**Table 5:** AI studies performed in lung ultrasound (US) for detection of pulmonary diseases.

Author/ Paper	Year of Publication	Field of Application	Dataset	AI Type	Results
Roy et al. [99]	2020	US: Multiclass—COVID-19 pneumonia - Severity - covid-19 markers	LUS	CNN	- Video processing - 96% accuracy
Tsai et. al. [96]	2021	US: BINARY—normal versus abnormal (bronchiolitis, bacterial pneumonia)	-5907 images from 33 healthy infants -3286 images from 22 infants with bronchiolitis -4769 images from 7 children with bacterial pneumonia	CNN	-Ablation study - 91.1% accuracy
Muhammad and Hossain [97]	2021	US: Multiclass—COVID-19, Pneumonia	POCUS (LUS Dataset comprising COVID-19, NON-COVID-19 and HEALTHY CASES	CNN	- Feature analysis - 92.5% accuracy
Barros et al. [92]	2021	US: Multiclass—COVID-19 Pneumonia	LUS	Hybrid CNN, LSTM	- Video processing - Ablation study - 93% accuracy
Diaz-Escobar et al. [93]	2021	Multiclass—COVID-19, Pneumonia, Healthy	POCUS -3326 pulmonary US frames	CNN	- Ablation study - 89.1% accuracy
Magrelli et al. [100]	2021	US: Multiclass—Healthy, Bronchiolitis, Bacterial Pneumonia Lung disease in children	Collected from Agostino Gemelli University Hospital	CNN	- Feature analysis - 97.75% accuracy
Dastider et al. [95]	2021	Severity prediction	COVID-19 LUS Database (ICLUSDB)	CNN, LSTM,	- 79.2% accuracy
Bhandari et al. [81]	2022	US: Multiclass-COVID-19, Pneumonia, Tuberculosis	Kermany, Chest X-ray (Covid-19 & Pneumonia) TUBERCULOSIS (TB) CHEST X-RAY DATABASE	CNN	- XAI - 94.31 accuracy
Ebadi et al. [90]	2022	US: Multiclass—A-lines, B-lines, consolidation, or pleural effusion	LUS	CNN	- 3D - Feature analysis - 90% accuracy
Shea et al. [72]	2023	US: Multiclass—Pleural effusion and B Lines (single and merged)	Collected from patients of all ages in Nigeria and China.	CNN, LSTM	- Video processing - XAI - 90% accuracy
Li et al. [98]	2023	US: Binary—Negative, Positive	Clinical dataset collected from 8 U.S. clinical sites between 2017 and 2020	CNN, LSTM	- 93.6% accuracy

CNN—the convolutional neural network; LSTM—long short-term memory; LUS—lung ultrasound; US— ultrasound; XAI—explainable artificial intelligence.

Acute Respiratory Distress Syndrome (ARDS) is a life-threatening lung condition characterized by severe hypoxemia, lung inflammation, and fluid build-up in the alveoli. AI-based clinical decision support (CDS) tools (Table 6) transform intensive care unit (ICU) management by predicting the risk of ARDS, helping ICU triage and helping with early intervention. DL analyzes real-time EHRs, vital signs, blood gases, and ventilator parameters, predicting ARDS up to 12–24 h before onset [39,46,101,102,103,104]. For example, Lam et al. [101] developed a recurrent neural network (RNN) model capable of identifying ARDS risk from continuously updated ICU data. Similarly, Desautels et al. [105] have demonstrated that early prediction using ML significantly improves early intervention opportunities in critically ill patients. These models outperform traditional scoring systems by integrating complex, time-dependent physiological data streams [39,102,103,104].

#### 2.1.2. CXR, CT Scan, and LUS: Pulmonary Nodules and Lung Cancer Screening

AI-based algorithms analyze (segment and categorize) CXR and CT scans for nodule detection and classification [14,23,52,59]. Notably, Setio et al. [106] and Ardila et al. [107] have demonstrated the effectiveness of DL systems in detecting and characterizing lung nodules with performance comparable to expert radiologists. The high-power quantitative analysis of fine structural image alterations, as shown in the work by Hawkins et al. [108], could be used to predict the probability of malignancy and the anticipated tumor kinetics [14]. Ground-glass opacities (GGOs) and subsolid nodules are challenging for human radiologists, as noted by Choi et al. [109]. AI-enhanced Computer-Aided Detection (CAD) systems can help radiologists detect lung nodules with greater accuracy. In quantitative imaging, AI-driven radiomics extracts advanced imaging features from CT scans to identify subtle disease patterns, as shown by Aerts et al. [110] (Table 4; Figure 6).

**Figure 7:**
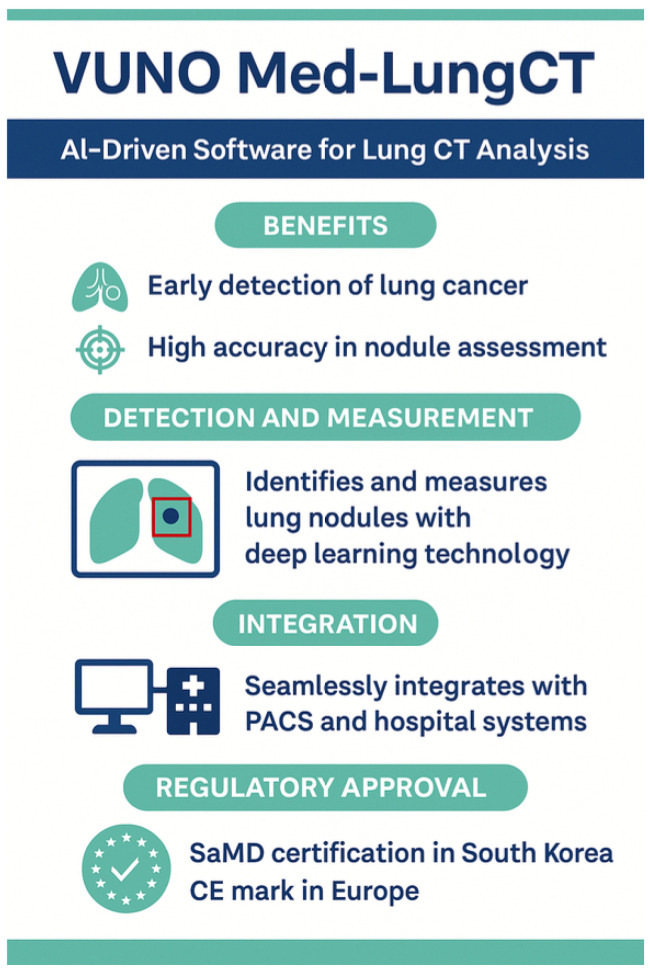
Example of AI-powered medical software (SaMD) in lung cancer detection.

A high-yield niche for AI imaging is cancer detection and characterization. LC is the leading cause of cancer-related deaths, having the highest morbidity worldwide [22,111,112]. AI-powered risk assessment models (for example, PanCan and Brock models) integrate CT imaging with clinical data (smoking history, age, genetics, etc.) to identify high-risk individuals for LC and guide personalized screening schedules, reducing unnecessary radiation exposure [14,21,23,47,59,104,106,113]. AI uses DL-CNN to analyze low-dose CT (LDCT) scans to identify nodules, define risk assessment (based on features such as size, shape, texture, and density) and reduce false negatives; LDCT is the gold standard for LC screening in high-risk individuals (e.g., heavy smokers) [21,23,58,59,65,112]. VUNO Med-Lung-CT is an example of AI-powered medical software classified as SaMD (Figure 7). DeepLung, LUNA16, and qCT-Lung detect and classify lung nodules in high-resolution CT (HRCT) scans [60].

**Figure 8:**
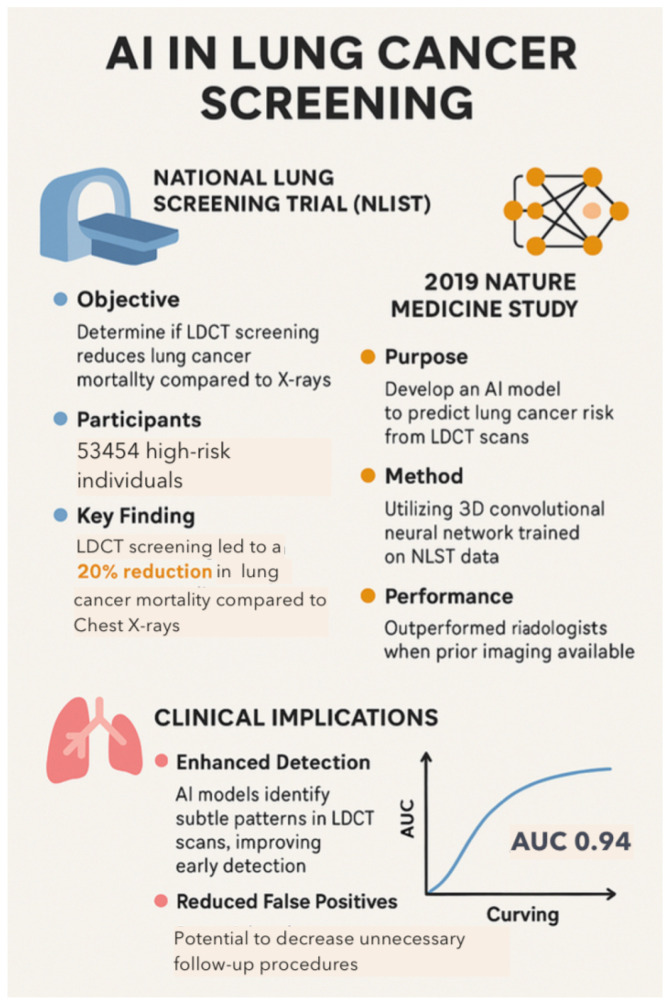
*Nature Medicine* study (2019): a DL model for LDCT analysis. The purpose of the study was to develop an AI model to predict LC risk from LDCT scans, utilizing a 3D CNN trained on NLST data. AUC: area under the curve (for cancer prediction).

A 2019 study published in *Nature Medicine* used a DL model trained on National Lung Screening Trial (NLST) data to analyze LDCT scans for LC screening. The study showed improved detection and a reduction in false positives and helped radiologists with decision making [21,47,59,106,114]. The NLST and AI-CAD systems revealed that AI outperforms radiologists in lung nodule detection, with 94% accuracy for AI in detecting malignant lung nodules (Figure 8). Google’s AI system, trained on NLST data, automatically analyzes LDCT scans, increasing the detection rate of lung nodules by 5% while reducing false positives by 11%. AI algorithms like Google’s DeepMind and Qure.ai analyze LDCT scans with 95% accuracy [21,23,74]. By improving early detection, AI-powered LDCT screening improved early-stage LC detection rates by 50%, increased 5-year survival rates, and significantly reduced mortality and unnecessary biopsies (by 30%) [20,23,106].

AI is also being integrated into Lung-RADS (Lung CT Screening Reporting and Data System), improving lung nodule classification, malignancy risk assessment, and follow-up recommendations in LDCT LC screening; it classifies nodules into categories based on their likelihood of being cancerous and suggests watchful waiting, short-term follow-up, or biopsy based on risk levels. AI-based Lung-RADS automation (e.g., Qure.ai and VUNO Med-LungCT AI) improves the efficiency of the radiologist, reducing false positives by 30% and improving early LC detection by 25% [65,113]. Rule-based AI systems have achieved varying degrees of clinical value in LC: diagnosis, staging, treatment, and prognosis. Rule-based AI systems are a type of AI that makes decisions using a set of predefined rules, manually created by experts based on domain knowledge [22,24,28,47,54,63,115,116].

#### 2.1.3. MRI

Although MRI is not the first-line imaging modality for lung diseases due to challenges with lung air–tissue contrast, AI is helping overcome these limitations, making MRI more viable for the diagnosis and monitoring of lung diseases (pulmonary fibrosis, LC, and vascular diseases) [60,69,73]. AI-based reconstruction improves signal-to-noise ratio (SNR) and reduces artifacts, allowing for precise lung and lesion segmentation. MRI-applied AI models are capable of analyzing ventilation, perfusion, and lung biomechanics. AI models process 4D flow MRI to analyze pulmonary artery blood flow and detect pulmonary embolism (PE) and pulmonary hypertension (PH).

AI-enhanced MRI is being explored as an alternative to LC screening in radiation-sensitive populations. AI models improve the detection of lung tumors and metastasis in MRI, particularly in hybrid positron emission tomography (PET-MRI), and also increase automated nodule detection and characterization, reducing false positives [58].

AI is improving and making lung magnetic resonance imaging a viable alternative to CT for certain lung diseases, reducing radiation exposure. However, technical and cost challenges must be overcome, and the need for more MRI datasets for robust training must be met [73].

#### 2.1.4. Bronchoscopy

DL algorithms are able to analyze abnormal structures in tissues or cells, improving the early detection of lung nodules and lung diseases, such as LC [21,22,23,48,54,59,65,66,80,81,82,106,109]. In bronchoscopy, DL has allowed in recent years for the highly accurate identification and labeling of bronchial segments solely from intraluminal bronchial images [109].

AI-guided robotic bronchoscopy integrates AI algorithms (such as DL) with robotic systems to navigate complex bronchial pathways, allowing for the accurate targeting of peripheral lung nodules (PLNs), increased diagnostic yield, and safety profile, enabling the accurate sampling of small and difficult-to-reach nodules [22,23,60,109,117]. This facilitates the early detection of LC and reduces the risk of complications associated with conventional biopsy techniques. Ion Endoluminal System (by Intuitive Surgical) is a notable example of a robotic bronchoscopy platform that integrates AI and shape-sensing technology. In the PRECISE clinical study (69 patients with PLNs), this technology has demonstrated a 83% diagnostic yield. The sensitivity to detect malignancy in biopsy samples ranged from 84% to 88%. The ideal candidates for this system are patients with small PLNs (less than 3 cm), especially those without a visible bronchus sign on CT imaging. In cadaver studies, the Ion Endoluminal System achieved a rate of lesion localization of 100%, surpassing electromagnetic navigation (EMN) by 15% and conventional methods by 35% and achieving a successful biopsy in 80% cases: a significant improvement over EMN alone (45%) and conventional methods (25%) [21,118].

The MONARCH platform is an AI-integrated robotic-assisted bronchoscopy, which also detects small, hard-to-reach lung tumors [118]. Institutions like the UC Davis Medical Center have embraced robotic-assisted bronchoscopy, performing a significant number of procedures [21,118]. Differential diagnosis between benign and malignant lesions has also been explored, using AI analysis of endobronchial ultrasound images (EBUS) [63,119].

### 2.2. AI-Liquid Biopsy in Lung Cancer Screening

AI analyzes circulating tumor DNA (ct-DNA), exosomes, and microRNAs from blood samples [69,116,119,120]. AI-driven liquid biopsy (e.g., Grail’s Galleri test) identifies LC signals years before clinical symptoms occur and the imaging shows the tumor [74,120,121]. For example, Cancer Likelihood in Plasma (CliP), developed by researchers at Stanford University, integrates various genomic features within an ML framework to detect early-stage LC from blood plasma. By distinguishing tumor-derived ctDNA from other sources, such as clonal hematopoiesis, CLiP enhances the specificity of early cancer detection.

### 2.3. AI in Pulmonary Function Testing

ML and DL models are also being integrated into pulmonary function tests (PFTs) to enhance the detection of lung diseases [37,54,68,83,122]. PFTs are essential to diagnosing and managing respiratory diseases, playing an important role in the decision tree for surgery in LC [123,124]. 8012ArtiQ.PFT 1.5.0 is an AI software application designed to automate the interpretation of PFTs, and its effectiveness has been demonstrated in several studies [54,122,123,124].

ArtiQ.PFT analyses spirometry, lung volumes, and DLCO, along with patient characteristics (age, smoking history, sex, and height), to generate a comprehensive report in less than one second. The software integrates smoothly into existing clinical workflows, such as Vyaire Medical’s SentrySuiteTM, allowing for immediate AI support in the same location as the PFT results.

AI models require high-quality, standardized PFT data for accurate predictions. AI must seamlessly integrate PFT data with EHR and must account for age, ethnicity, and comorbidities affecting lung function [68,122,123]. AI-enabled home spirometry will allow for the real-time monitoring of lung function [67].

## 3. AI Applications in Treatment

### 3.1. AI in Thoracic Surgery

Due to its high reliance on imaging and complex surgical decisions, AI is increasingly trying to enter TS, where it is expected to play a key role in diagnosis, management, and surgical decision making in the years to come [28,55,107]. Little has been published specifically in TS, but AI is becoming more prevalent in the surgical sphere [21,74,102,118,120,125,126,127,128,129,130]. Table 7, Table 8 and Table 9 show some studies of AI in preoperative, intraoperative, and postoperative TS, respectively.

**Table 7:** Preoperative AI studies in thoracic surgery.

Author/ Paper	Year of Publication	Field of Application	Dataset	AI Type	Results
Esteva et al. [131]	2002	Assessment of surgical risk in patients undergoing pulmonary resection Prediction of postoperative outcomes in lung resections	96 clinical and laboratory features from each one of 141 patients who underwent lung resection (retrospectively collected)	Neural network	NN can integrate results from multiple data predicting the individual outcome for patients, rather than assigning them to less-precise risk group categories
Santos-Garcia et al. [132]	2004	To propose an ensemble model of ANNs to predict cardio-respiratory morbidity after pulmonary resection for NSCLC	-Prospective clinical study based on 489 NSCLC operated cases. -An artificial neural network ensemble was developed using a training set of 348 patients undergoing lung resection between 1994 and 1999	Artificial neural network	- ANN ensemble offered a high performance to predict postoperative cardio-respiratory morbidity
Naqi et al. [133]	2018	To develop a multistage segmentation model to accurately extract nodules from lung CT images. Lung nodule segmentation method.	-Publicly available dataset, namely lung image database consortium and image database resource initiative	Support vector machine	- 99% accuracy -98.6% sensitivity -98.2% specificity -3.4% false positives per scan
Bolourani et al. [129]	2021	To identify risk factors for respiratory failure after pulmonary lobectomy	National (Nationwide) Inpatient Sample for 2015 was used to establish the model. -A total of 4062 patients who underwent pulmonary lobectomy	Random forest ML	- The first ML-model, with high accuracy and specificity, is suited for performance evaluation, - The second ML-model, with high sensitivity, is suited for clinical decision making
Salati et al. [130]	2021	To verify if the application of an AI analysis could develop a model able to predict cardiopulmonary complications in patients submitted to lung resection	-Retrospectively analyzed data of patients submitted to lobectomy, bilobectomy, segmentectomy and pneumonectomy (January 2006-December 2018) -1360 patients (lobectomy: 80.7%, segmentectomy: 11.9%, bilobectomy 3.7%, pneumonectomy: 3.7%	Extreme gradient boosting	XGBOOST algorithm generated a model able to predict complications with an area under the curve of 0.75
Chang et al. [102]	2021	Prediction of staged weaning from ventilator after lung resection surgery	-Retrospectively collected EHRs of 709 patients who underwent lung resection between 1 January 2017 and 31 July 2019	Multiple ML algorithms	The AI model with Naïve Bayes Classifier algorithm had the best testing result and was therefore used to develop an application to evaluate risk based on patients’ previous medical data, to assist anesthesiologists, and to predict patient outcomes in pre-anesthetic clinics
Sang et al. [134]	2025	Comparison of the application effects of AI software and Mimics software for 3D reconstruction in thoracoscopic anatomic segmentectomy. -Multiclass—COVID-19, Pneumonia, Normal, Other LUS TD-CNNLSTM-Lung-Net	Retrospective cohort study -168 patients divided into 3 groups: AI group (*n* = 79), Mimics group (*n* = 53), and control group without 3D reconstruction (*n* = 36)	-AI software -Mimics Software	- Preoperative 3D reconstruction, whether using AI or Mimics, resulted in shorter operation times, reduced intraoperative bleeding, and shorter postoperative hospital stays compared to the control group. - AI software demonstrated comparable efficacy to Mimics, with the added advantage of reducing the workload on clinicians due to its automated processes. - Video processing - 3D model - Ablation study - Feature analysis - XAI - 96.57% accuracy

AI—artificial intelligence; ANN—artificial neural network; AUC—area under the curve; CNN—convolutional neural networks; CT—computed tomography; EHR—electronic health records; LSTM—long short-term memory; ML—machine learning; NN—neural networks; NSCLC—non-small cell lung cancer; XAI—explainable artificial intelligence.

**Table 8:** Intraoperative AI studies in thoracic surgery.

Author/ Paper	Year of Publication	Field of Application	Dataset	AI Type	Results
Shademan et al. [117]	2016	Feasibility of supervised autonomous robotic soft tissue surgery in na open surgical setting. Demonstrate in vivo supervised autonomous soft tissue surgery in an open surgical setting, enabled by a plenoptic three-dimensional and near-infrared fluorescent (NIRF) imaging system and an autonomous suturing algorithm.	Ex vivo porcine intestines	Smart Tissue Autonomous Robot	The outcome of supervised autonomous procedures is superior to surgery performed by expert surgeons
Cho et al. [135]	2018	To enhance the accuracy of gesture recognition for contactless interfaces	—Used 30 features including finger and hand data, which were computed selected, and fed into a multiclass support vector machine (SVM), and Naïve Bayes classifiers: to predict and train five types of gestures including hover, grab, click, one peak, and two peaks.	Support vector machine classifier and Naïve Bayes classifier	Overall accuracy of the five gestures was 99.58% and 98.74%, on a personal basis using SVM and Naïve Bayes classifiers
Wang et al. [57]	2018	Objective skill evaluation in robotic-assisted surgery	-Perform experiments on the public minimally invasive surgical robotic dataset, JHU-ISI Gesture and Skill Assessment Working Set (JIGSAWS).	CNN DL	- Achieved competitive accuracies of 92.5%, 95.4%, and 91.3%, in the standard training tasks: suturing, needle-passing, and knot-tying
Fard et al. [136]	2018	To build a classification framework to automatically evaluate the performance of surgeons with different levels of expertise - Automated robotic-assisted surgical evaluation	8 global movement features are extracted from movement trajectory data captured by a da Vinci robot for surgeons with 2 levels of expertise—novice and expert	Multiple ML algorithms	-The proposed framework can classify surgeons’ expertise as novice or expert with an accuracy of 82.3% for knot tying, and 89.9% for a suturing task
Dai et al. [45]	2019	To develop and validate a novel grasper-integrated system with biaxial shear sensing and haptic feedback to warn the operator prior to anticipated suture breakage	Novice subjects (*n* = 17) were instructed to tighten 10 knots, five times with the Haptic Feedback System (HFS) enabled, five times with the system disable	Biaxial haptic feedback system sensors were integrated with a da Vinci robotic surgical system.	This system may improve outcomes related to knot tying tasks in robotic surgery and reduce instances of suture failure while not degrading the quality of knots produced
Ershad et al. [137]	2019	To propose a sparse coding framework for automatic stylistic behavior recognition in short time intervals using only position data from the hands, wrist, elbow, and shoulder - Evaluation of technical skills in robotic surgery	- A codebook is built for each stylistic adjective using the positive and negative labels provided for each trial through crowd sourcing. Sparse code coefficients are obtained for short time intervals (0.25 s) in a trial using this codebook. A support vector machine classifier is trained and validated through tenfold cross-validation using the sparse codes from the training set	Support vector machine	- The proposed dictionary learning method can assess stylistic behavior performance in real time
Wu et al. [114]	2021	Effectiveness in localizing small pulmonary nodules during thoracic surgery	30 patients	AI-based semi-automatic and high-precision pulmonary 3D reconstruction system - DL to segment and reconstruct tumors, lobes, bronchi, and vessels.	- Significantly reduced reconstruction time compared to conventional tools like Mimics - Maintaining high accuracy in identifying pulmonary nodules and anatomical structures.
Li et al. [128]	2022	Reconstruction system to assist Thoracic Surgery: - Proposed goals: enhance preoperative planning and intraoperative navigation	500 cases (Retrospective) 113 patients with lung cancer before surgery (Prospective) 139 patients scheduled to undergo lobectomy or segmentectomy	AI- assisted three-dimensional reconstruction (AI-3DR) (CNN)	- Automates the reconstruction process, significantly reducing the time required compared to manual methods. - Decrease reconstruction time from 30 min to approximately 5 min. - high accuracy in predicting affected lung segments - Operation time shortened by 24.5 min for lobectomy (*p* < 0.001) and 20 min for segmentectomy (*p* = 0.007).

**Table 9:** Post-surgical applications of AI in TS.

Author/ Paper	Year of Publication	Field of Application	Dataset	AI Type	Results
Chang et al. [102]	2021	Prediction of staged weaning from ventilator after lung resection surgery	-Retrospectively collected the EHRs of 709 patients who underwent lung resection	Multiple ML algorithms	The AI model with Naïve Bayes Classifier algorithm had the best testing result and was therefore used to develop an application to evaluate risk based on patients’ previous medical data, to assist aesthesiologists, and predict patient outcomes in pre-anesthetic clinics
Kana et al. [94]	2025	Improvement of postoperative documentation. Proposed goals: AI can generate post-operative notes more accurately than surgeons	158 cases from a tertiary referral centre	By utilizing computer-vision systems to observe robot-assisted surgeries	- AI produced narratives with fewer discrepancies and significant errors - AI enhances surgical documentation and reduces the workload on surgeons. —Overall accuracy is higher for AI operative reports as compared to surgeon operative reports (87.3% versus 72.8%).

AI—artificial intelligence; EHR—electronic health records; ML—machine learning.

#### 3.1.1. Preoperative AI Applications

AI is revolutionizing TS by improving risk stratification, 3D reconstruction, and preoperative decision making.

**Figure 9:**
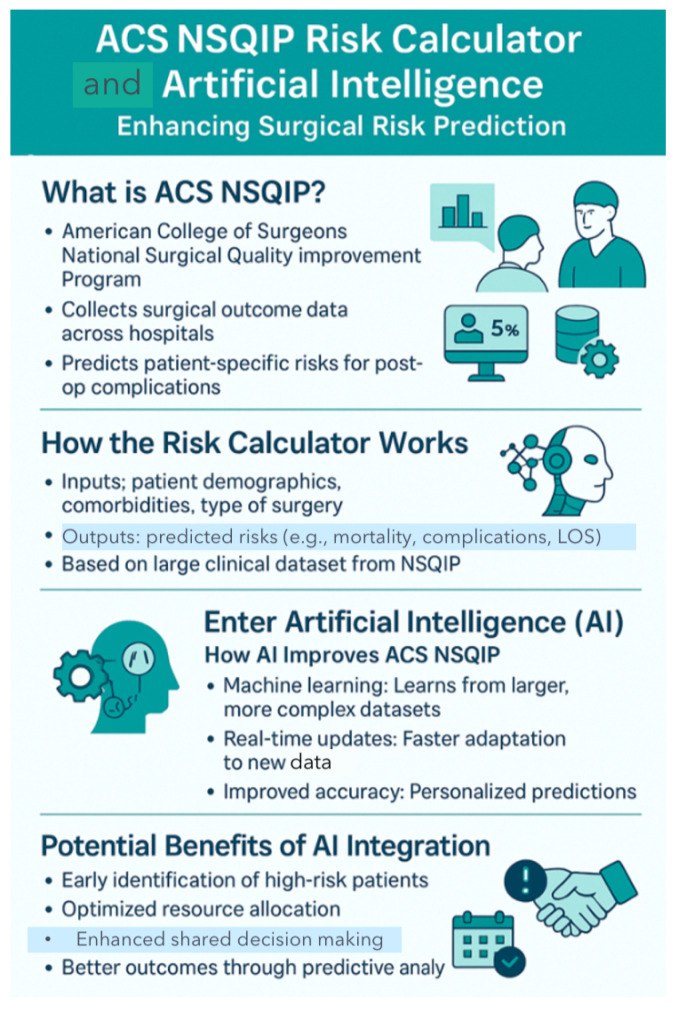
ACS NSQIP risk calculator: enhancing surgical risk prediction.

AI-based preoperative risk prediction helps predict and prevent complications after thoracic surgery: The ACS NSQIP Surgical Risk Calculator is an AI-enhanced tool designed by the American College of Surgeons (ACS) to predict the likelihood of complications after TS (Figure 9). As demonstrated by Bilimoria et al. [138], and further explored by Khuri et al. [139], this tool has been instrumental in guiding preoperative optimization strategies, such as identifying high-risk patients who may benefit from ICU monitoring, involving cardiology for perioperative planning, selecting appropriate prophylactic antibiotics, and tailoring intraoperative techniques and postoperative drain management. It also helps optimize discharge planning Enhanced Recovery After Surgery (ERAS) [21,29,37,55,104,118]. In this way, AI can guide surgeons in applying interventions such as smoking cessation, pulmonary rehabilitation, nutrition support, and early mobilization [21,118,129,131,132].

AI predicts postoperative lung function based on preoperative imaging and PFTs. AI models can predict which LC patients will benefit from surgery versus stereotactic body radiation therapy (SBRT) [140]. AI-based CDS assists in selecting the optimal surgical approach (open surgery, VATS, or RATS) and optimizes anesthesia and ventilation strategies based on predictions of lung function [22,118,134]. Three-dimensional AI-enhanced imaging helps thoracic surgeons to adjust surgical plans based on risk lung resections, in minimally invasive procedures, minimizing risks and providing better outcomes [104,128,134]. AI-powered 3D modeling creates patient-specific virtual simulations for VATS and RATS.

Risk stratification does not predict long-term survival or cancer recurrence. Personalized AI for rare thoracic procedures is still evolving, and future AI tools may combine EHR data with wearable monitoring for dynamic risk prediction [104,118,130].

#### 3.1.2. Intraoperative AI Applications

**Figure 10:**
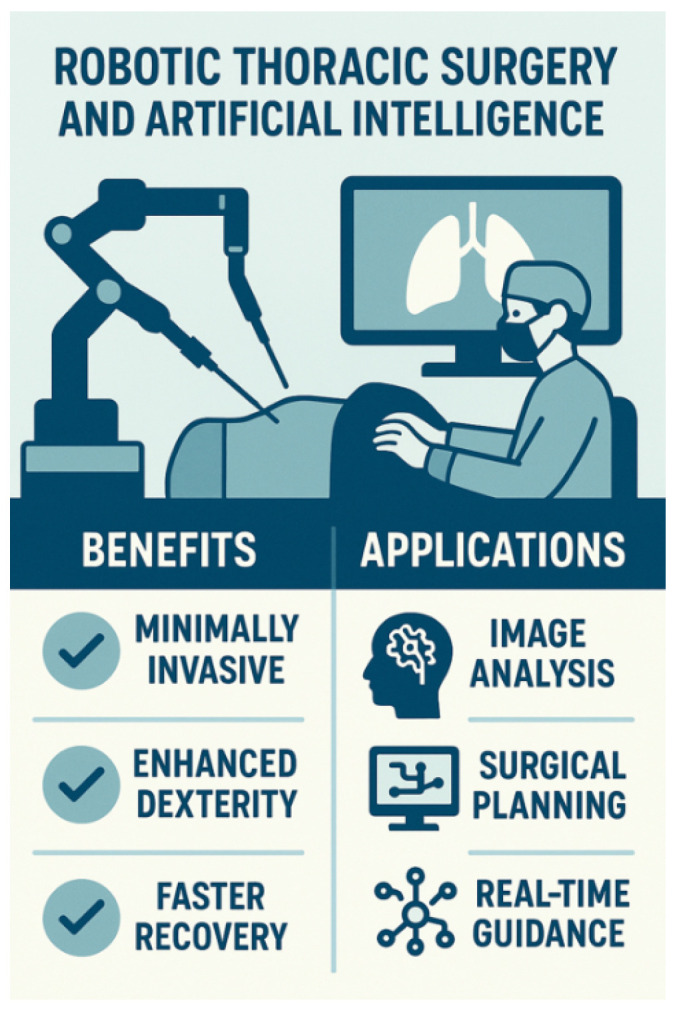
AI-assisted robotic thoracic surgery.

Applications in TS include surgical navigation and robotics [28,45,49,55,56,57,69,103,117,135,136,137] (Figure 10). AI-driven RATS integrates ML, real-time imaging, and robotic precision, improving dexterity, visualization, and control and leading to reduced complications and faster recovery [45,49,50,55,117,121,135,136,137]. Table 10 explains the applications of AI algorithms in RATS.

AI-powered robotic systems (Da Vinci Surgical System, Monarch, etc.) are revolutionizing thoracic procedures. AI helps plan and assist complex surgeries, providing 4K 3D visualization and tremor-filtered robotic precision for lung tumor resections [28,55,117]. During complex thoracic resections, AI supports intraoperative surgical decisions by enhancing visualization, anatomical orientation, and real-time feedback. AI systems integrate preoperative 3D imaging (CT, MRI, and PET) with intraoperative video and sensor data to guide anatomical dissection, identify critical structures (e.g., vessels, bronchi, and nerves), and monitor the surgical field in real time. AI algorithms refine instrument movement precision, minimizing surgical trauma, and reducing the risk of vascular injuries and prolonged air leaks (PALs) [134,136] (Table 8 and Table 11). For example, AI-powered augmented reality (AR) overlays pre-segmented anatomical maps onto the surgical view, allowing the surgeon to anticipate variations and reduce risks to adjacent organs [28,46,56,117,118]. In trials by Li et al. [128], AI-assisted anatomical guidance reduced operative time and improved the identification of segmental bronchi and vessels, particularly in complex segmentectomies or anatomical variants.

AI also enables real-time tumor tracking and margin analysis. Algorithms can detect tumor boundaries using intraoperative imaging and machine vision, helping ensure complete resection while preserving healthy lung parenchyma. As demonstrated by Kanavati et al. [65], this improves surgical precision during sublobar resections and significantly reduces the risk of positive surgical margins: a major prognostic factor in oncologic outcomes [56,117,136]. Studies such as Lin et al. [141] report that AI-guided resections result in a reduction of up to 30% in positive margins, especially in early-stage NSCLC and small subsolid lesions [55,56,118].

All these features have been shown to improve surgeon confidence and precision, reduce unplanned conversions to open surgery, and minimize intraoperative errors [21,50,55]. AI is being incorporated into surgical training programs, providing simulation-based education, performance assessment, surgical proficiency, and patient safety. As described by Madani et al. [142] and Hashimoto et al. [143], AI-powered simulators and video analysis tools allow trainees to refine their techniques in controlled, high-fidelity environments while enabling instructors to assess technical skills and cognitive decision making with greater objectivity [49,55,56,121].

Several AI tools have received regulatory approval and are currently integrated into thoracic surgical and diagnostic workflows. In the intraoperative setting, the Da Vinci Surgical System (by Intuitive Surgical) is FDA-approved, and while not AI-native, it is now integrating AI features such as automated anatomical annotation and tremor filtering in next-generation models [21,50,55,121]. The Monarch robotic platform (Auris Health) is also FDA-cleared for bronchoscopy, with AI-assisted navigation capabilities [21,49,50]. While many AI applications in TS remain under investigation or are in early clinical integration (e.g., AR-based navigation and intraoperative margin prediction), the tools above represent AI technologies with regulatory validation currently shaping real-world practice [55,121].

Despite its growing utility, AI in TS has limitations, particularly in the intraoperative context. One of the foremost challenges is the dependence on high-quality imaging [28,117]. AI-based decision support tools (whether for navigation, segmentation, or margin detection), require consistently clear and standardized imaging inputs (e.g., CT, intraoperative video, and fluorescence imaging). Variability in imaging resolution, patient anatomy, or intraoperative conditions (e.g., blood, motion, and lighting) can reduce the accuracy and reliability of AI predictions. Moreover, unlike experienced surgeons, AI systems currently lack tactile perception, which is a key factor in differentiating tissue textures, assessing adhesions, or identifying tumor invasion during dissection. This absence of haptic feedback may limit AI’s role in delicate procedures such as sleeve resections or extrapleural pneumonectomies, where tissue feel can influence real-time decisions. Other concerns include algorithm generalizability, as many AI models are trained on narrow datasets from specific populations or institutions, limiting their robustness across diverse patient cohorts. Additionally, black-box decision making remains a barrier to full trust and adoption. Lastly, legal liability, data security, and surgeon oversight remain unresolved in many health systems, raising ethical and professional concerns about over-reliance on AI in high-stakes environments. A balanced understanding of these limitations is essential to integrating AI safely and effectively into thoracic surgical workflows [50,56,144].

**Table 10:** AI algorithms applications in RATS: surgical planning, precision, navigation, and real-time guidance.

AI Technology/Algorithm	Description	Application in Robotic Thoracic Surgery
AI-Driven Robotics	Refers to robotic systems that use AI to enhance automation, improve precision, and assist in decision-making. AI algorithms analyze patient data to optimize surgical planning and execution.	Robotics like the da Vinci Surgical System and Ion Endoluminal System are AI-driven, assisting with tasks like nodule localization, resection planning, and enhancing the surgeon’s ability to navigate complex anatomy.
Convolutional Neural Networks (CNN)	A deep learning model primarily used for image recognition and classification tasks, especially in medical imaging.	Applied in robotic thoracic surgery for tumor detection, classification of lung lesions, and segmentation of anatomical structures in CT scans and MRI.
Reinforcement Learning (RL)	AI learns through trial and error, receiving rewards or penalties based on its actions. In surgery, it improves decision-making and optimizes robotic arm movements.	RL can improve robot control systems in surgery, learning optimal movements to improve precision and reduce errors during complex thoracic surgeries.
Support Vector Machines (SVM)	A machine learning model used for classification tasks by finding the best boundary between classes.	Used for classifying lung lesions based on their radiographic features, helping in preoperative planning and diagnosis.
Random Forest	An ensemble method that uses multiple decision trees for classification and prediction.	Applied in predicting surgical outcomes or complications, or for improving the classification of lesions based on imaging features.
K-Nearest Neighbors (K-NN)	A machine learning algorithm that classifies data points based on their proximity to other points in feature space.	Used for comparing lung lesions to a database of known cases, assisting in the classification of lesions or abnormal tissue.
Generative Adversarial Networks (GANs)	AI where two networks compete to generate synthetic data. GANs can create realistic images or datasets.	Used to generate high-quality medical images for training AI models or simulating surgical scenarios, improving robotic system training.
Artificial Neural Networks (ANN)	Computational models inspired by the human brain, often used for prediction, classification, and decision-making.	ANN is used in robotic systems to predict the outcome of surgery, assess patient data, or assist in real-time decision-making during procedures.
Bayesian Networks	Probabilistic graphical models that represent variables and their conditional dependencies.	Used for predicting surgical risks, complications, or likely patient outcomes based on preoperative data.
AI-Augmented Reality (AR)	Combines AI with AR to overlay digital information onto the real-world view.	In thoracic surgery, AR can overlay 3D imaging data (e.g., from CT or MRI scans) onto the surgeon’s view, guiding robotic instruments and improving navigation to target tissues.
AI-Enhanced Image-Guided Surgery	Uses AI to interpret real-time imaging data to guide the surgical robot’s actions.	In thoracic surgery, AI-driven systems analyze live images (e.g., CT, MRI, or fluoroscopy) to help surgeons navigate and perform delicate procedures such as biopsy or tumor resection.
Natural Language Processing (NLP)	AI techniques that understand and process human language from text or voice data.	Used in robotic surgery to analyze patient records, surgical notes, or voice commands to assist in planning or executing surgery. NLP can also help in analyzing large datasets from patient histories.
AI-Powered Robotic Arm Control	AI algorithms that assist in the control of robotic arms, ensuring high precision during surgery.	Used in systems like the da Vinci to provide real-time feedback, adjust the robot’s movements, and improve surgical precision, especially in complex thoracic procedures.
AI in Patient Monitoring	AI algorithms monitor and analyze patient vitals, detecting abnormalities in real-time.	During robotic surgery, AI helps track patient vitals (e.g., heart rate, oxygen levels), ensuring the safety of the patient by alerting the surgical team to any significant changes.
Autonomous Surgical Systems	AI systems capable of performing portions of surgery with little to no human intervention.	Some AI-powered robotic systems may assist with tasks such as tissue resection, suturing, and even autonomously guiding tools through specific parts of the thoracic anatomy.
AI in Surgical Planning	AI algorithms that analyze patient data to suggest the best surgical approach and plan.	AI can analyze CT, MRI, and biopsy data to recommend the optimal approach for thoracic surgery, including which surgical instruments to use and the most efficient path to reach the target tissue.

**Table 11:** Other published studies of AI in RATS.

Study	Authors	Year	AI Application	Quantitative Results
**Artificial intelligence in thoracic surgery: a narrative review**	Bellini V, et al. [55]	2022	Review of AI applications across thoracic surgery	Highlights AI’s role in enhancing perioperative evaluations, decision-making, surgical performance, and operating room scheduling. Specific quantitative data not provided.
**Evaluation of the Postoperative Nursing Effect of Thoracic Surgery Assisted by Artificial Intelligence Robot**	Hu et al. [145]	2021	AI-assisted postoperative nursing in thoracic surgery	Utilized the Da Vinci robotic system for lobectomy. Reported operation times ranging from 62 to 225 min and blood loss between 70 to 300 mL. No intraoperative blood transfusions were required.
**Artificial intelligence–assisted augmented reality robotic lung surgery: Navigating the future of thoracic surgery**	Sadeghi AH, et al. [146]	2024	AI-assisted augmented reality in robotic lung surgery	Achieved a 99.58% overall accuracy in gesture recognition using support vector machine and Naïve Bayes classifiers. Developed a deep learning framework for surgical skill assessment, achieving accuracies of 92.5%, 95.4%, and 91.3% for suturing, needle-passing, and knot-tying tasks, respectively.

#### 3.1.3. Postoperative AI Applications

AI improves postoperative care in the ICU, allowing for the early detection of complications, the optimization of rehabilitation, and the improvement in long-term outcomes [101,130] (Table 9). AI-driven postoperative monitoring and predictive analytics are transforming recovery after TS by continuously analyzing real-time patient data from wearables, ICU monitors, and EHRs [46,101,103].

ML models, trained on historical data from thousands of cases, can predict pulmonary complications (e.g., pneumonia, atelectasis, respiratory failure, and PALs), providing early interventions. A PAL, defined as an air leak with a duration of >5 days, is the most common complication after lung surgery. Recent studies by Brunelli et al. [104] have shown that AI models can predict PALs by integrating preoperative CT-derived lung density metrics with intraoperative findings, thereby supporting decisions around chest tube duration and reducing the risk of overtreatment or delayed discharge [3,46,129,132]. Other AI-guided strategies help adjust drainage management and early discharge planning.

AI-powered Early Warning Systems (EWSs) are transforming postoperative monitoring by predicting respiratory failure (RF), sepsis, and ICU deterioration in TS patients [67,101,102,126,127]. These systems analyze real-time vital signs (HR, oxygen saturation, RR, BP, etc.), laboratory values, and patient trends to provide early alerts, allowing for timely interventions. CLEW ICU, an FDA-cleared AI-powered EWS, predicts postoperative RF (often before clinical symptoms occur), nearly 8 h in advance, reducing stays in the ICU related to respiratory failure by 30% and allowing clinicians to initiate early interventions such as non-invasive ventilation (NIV) or high-flow oxygen therapy (HFOT), with improvement in survival rates and reduction in the severity of postoperative ARDS [25]. ML algorithms can help ventilator management in the ICU, optimizing respiratory support for critically ill patients [101,102]. AI-based extubation failure models have reduced unplanned reintubation rates by 20% in TS ICU [102,126,127].

AI-driven smart patches, pulse oximeters, and respiratory monitors follow patients after discharge, alerting physicians to deterioration [67]. AI also integrates home oxygen saturation monitoring and step count data to assess recovery and predict risk of rehospitalization [68]. In this way, AI-powered wearable biosensors (e.g., Current Health and Biofourmis) track oxygen desaturation and HR variability to detect postlobectomy complications before symptoms appear, performing remote patient monitoring [55,118]. In the future, Smart Wearables will perform continuous remote lung sound monitoring to detect early pneumonia.

AI algorithms in the ERAS protocols predict which LC patients need closer post-discharge follow-up to prevent readmissions [21,118]. AI evaluates preoperative risks, surgery type, and postoperative vitals to predict the risk of readmission at 30 days [104]. AI-driven postoperative lung function monitoring reduced readmission rates by 30% in postlobectomy patients.

AI also helps in personalized rehab planning, based on recovery speed and lung function trends. Emerging AI applications are transforming patient rehabilitation and monitoring. For example, Israeli medical centers have employed AI-driven therapies (e.g., video game therapy) to aid in patient recovery. These technologies offer innovative approaches to rehabilitation.

### 3.2. AI in Lung Cancer Treatment Planning

AI is transforming LC treatment planning not only by optimizing surgical decision making but also by improving tumor characterization and staging, radiation therapy planning, and personalized therapy selection [87]. In staging, AI-powered Lung-PET DL models and AI-guided TNM staging improved tumor staging accuracy by 20%, allowing for the identification of lymph nodes and distant metastases [63,74,86,87,116,120]. AI-driven radiomics extracts patterns from CT scans to predict tumor aggressiveness and response to treatment, guiding personalized therapy (surgery, radiation therapy, immunotherapy, etc.) [14,58].

AI allows for precision medicine in drug therapy. AI-driven models integrate imaging, pathology, and molecular data to enhance precision medicine and improve patient outcomes [74,87]. AI can optimize chemotherapy regimens and tailor immunotherapy or targeted therapies by analyzing complex multi-omics datasets. For instance, when AI integrates radiomics with genomic alterations, such as EGFR mutations or ALK rearrangements, it can accurately predict therapeutic responses and inform oncologists on whether a patient is more likely to benefit from EGFR inhibitors, ALK inhibitors, or immune checkpoint blockade therapies [74,87,140]. In studies such as those by Somashekhar et al. [147], AI-powered Tempus and IBM Watson Oncology improved targeted therapy selection by 35%, matching patients with the most effective treatments [24,63,64,87,115,116,119,120,130,131]. Some recent advances of AI in lung pathology are described in Table 12.

AI-driven radiotherapy increased LC survival rates by 20%; it automatically segments tumors and organs at risk (OARs) in radiation planning, with greater control and lower toxicity [121,140]. AI-based software such as Varian’s Ethos™ adjusts radiation doses in real time, personalizing therapy for patients with NSCLC (Non-Small Cell Lung Cancer) [140]. Additionally, AI-based Monte Carlo simulations, enhance dose calculation accuracy and minimize radiation-induced lung injury [144]. These models simulate complex physical interactions within tissue, optimizing treatment planning and reducing complications. AI-based adaptive radiation therapy (ART) also adjusts the dose distribution based on real-time tumor changes. In proton therapy, AI predicts the response of the tumor, adjusts the intensity of the beam for greater precision, and reduces radiation side effects by 30%, according to Kang et al. (2023) [140].

**Table 12:** Recent advances of AI in lung pathology.

Author	Primary Goals	AI technology	Main Goals	Results
Yu et al. [148]	To improve the prognostic prediction of lung adenocarcinoma and squamous cell carcinoma patients through objective features from histopathology images	Elastic net-Cox proportional hazards model	Prediction of the prognosis of lung cancer by automated pathology image features and thereby contribution to precision oncology	-Image features can predict the prognosis of lung cancer patients
Coudray et al. [26]	To train a deep convolutional neural network on whole-slide images obtained from The Cancer Genome Atlas to accurately and automatically classify them	DL-CNN	Detection of cancer subtype or gene mutations and mutation prediction from NSCLC histopathology	-DL models can assist pathologists in the detection of cancer subtype or gene mutations
Wei et al. [27]	To propose a DL model that automatically classifies the histologic patterns of lung adenocarcinoma on surgical resection slides	Deep neural network	Improvement of classification of lung adenocarcinoma patterns	All evaluation metrics for the model and the 3 pathologists were within 95% confidence intervals of agreement
Gertych et al. [25]	To a pipeline equipped with a CNN to distinguish 4 growth patterns of pulmonary adenocarcinoma (acinar, micropapillary, solid, and cribriform) and separate tumor regions from non-tumor	CNN	To assist pathologists in improving classification of lung adenocarcinoma patterns by automatically pre-screening and highlighting cancerous regions prior to review	-Overall accuracy of distinguishing the tissue classes was 89.24%
KanavatI et al. [65]	To train a CNN, using transfer learning and weakly-supervised learning, to predict carcinoma in Whole Slide Images	CNN	Development of software suites that could be adopted in routine pathological practices and potentially help reduce the burden on pathologists	-Differentiating between lung carcinoma and non-neoplastic

CNN—convolutional network; DL—deep learning; NSCLC— non-small cell lung cancer.

### 3.3. AI in Prognosis and Survival Prediction: Lung Cancer

AI-driven models assist oncologists in long-term patient management. AI is revolutionizing LC prognosis and survival prediction by integrating radiomics, genomics, pathology, and clinical data to generate highly accurate risk stratifications and outcome forecasts.

A notable advancement in survival is the development of the AI foundation model “Chief” by Harvard Medical School, trained on millions of whole-slide tissue images. It has achieved an accuracy of up to 94% in cancer detection and has the potential to predict survival rates, marking a significant step forward in AI-assisted medical diagnostics [87,149]. AI models applied to EHR data from >10,000 LC patients improved survival predictions by 40% compared with TNM (Tumor, lymph Node, Metastasis) staging alone [24,37,63,64,115,116,119,120]. DL models trained on SEER (Surveillance, Epidemiology, and End Results) leverage a rich, population-based dataset that includes variables such as patient demographics, tumour stage and characteristics, histology, treatment details, and outcomes, being able to identify complex patterns and interactions, by processing complex, high-dimensional data. These models can improve survival predictions (5-year survival rates for various cancers) [23]. AI-driven molecular prognostic models (Tempus, IBM Watson Genomics, etc.) improved LC survival rates by 25%. AI-powered prognostic models, such as DeepSurv, improved 5-year survival prediction accuracy by 30% compared with traditional staging alone [117]. AI predicts overall survival (OS) and disease-free survival (DFS) based on patient demographics, comorbidities, treatment response, and heterogeneity on CT/PET scans (by AI-driven radiomics) [27,39,74,87,120].

AI-powered histopathology image analysis detects aggressive tumour phenotypes and stromal interactions linked to poor prognoses [148]. AI-based radiomic signatures predicted LC recurrence with 85% accuracy [58]. AI-driven liquid biopsy analysis detects ctDNA. This non-invasive approach enables clinicians to monitor tumour dynamics in real time, detecting minimal residual disease and molecular relapse before clinical symptoms emerge, which allows for earlier and potentially more effective treatments. Digital polymerase chain reaction (dPCR) enhances the sensitivity of ctDNA detection, allowing for the identification of rare mutations associated with tumour recurrence. Regular blood tests for ctDNA offer a less invasive alternative to traditional tissue biopsies, reducing patient discomfort and risk [87].

## 4. Challenges, Ethical Considerations, and Future Directions of AI in Thoracic Surgery

### 4.1. Challenges and Ethical Considerations

**Figure 11:**
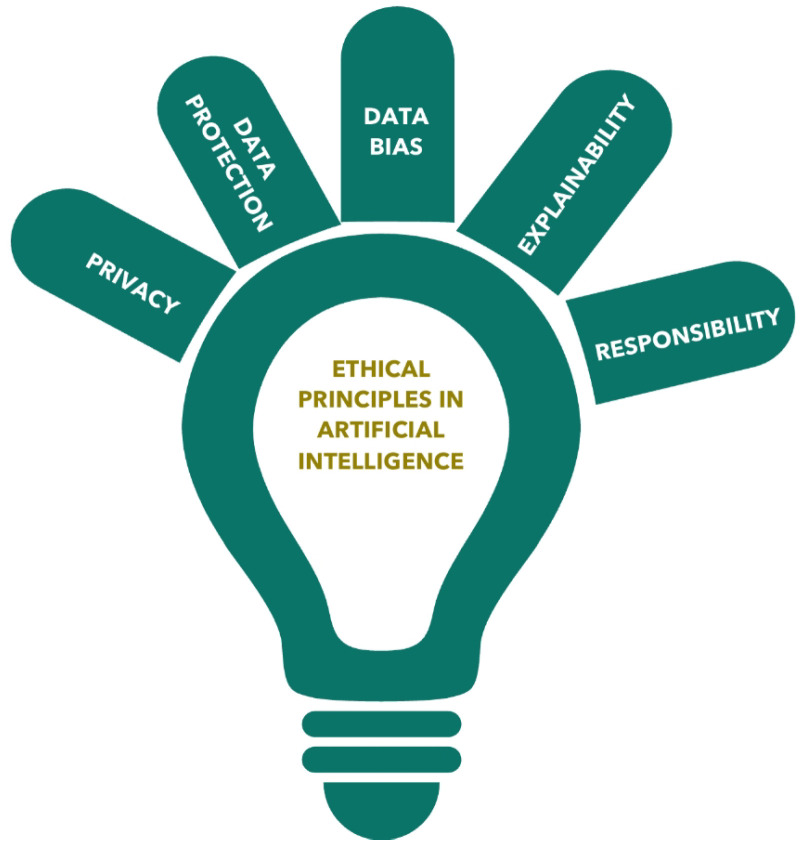
Ethical principles in artificial intelligence.

AI in TS holds significant promise for enhancing patient outcomes and operational efficiency. However, AI may also lead to job displacement, particularly in roles involving routine tasks, and presents several challenges with ethical considerations that need to be addressed [2,31,33,89]. The healthcare integration of AI technology, including SaMD, implies the insurance of patient safety, equity, and trust, on which privacy, data protection, data bias, explainability, and responsibility, rely [31,34,43,61] (Figure 11).

Organizations like the ITU-WHO Focus Group on AI for Health are working to create benchmarking processes to assess AI’s accuracy and safety in healthcare [31,33,61]. The International Medical Device Regulators Forum (IMDR) is an international group working to harmonize SaMD regulation: they develop guidelines and ensure safety and effectiveness. In Europe, regulations from the Medical Device Coordination Group (MDCG) clarify SaMD by risk, requiring specific assessments according to the device. EUDAMED was also designed to implement diagnostic Medical Devices [31,62].

Despite the promise of ML in TS, issues around patient data privacy and AI decision-making transparency, remain unresolved [28,32,33,43,53]. Handling sensitive patient information necessitates stringent data protection measures, such as compliance with regulations like the General Data Protection Regulation (GDPR) [8,31,34]. SaMD may collect and store sensitive patient data, which are easy to reproduce and vulnerable to remote access and manipulation. Healthcare organizations are increasingly targeted by cyberattacks aiming to exploit vulnerabilities in data storage. Hence, robust cybersecurity measures must be implemented to protect patient data [31,33]. A survey in the UK estimated that 63 per cent of the population is uncomfortable with sharing their personal data to improve AI technology, reflecting widespread concerns about data privacy and misuse [33].

**Figure 12:**
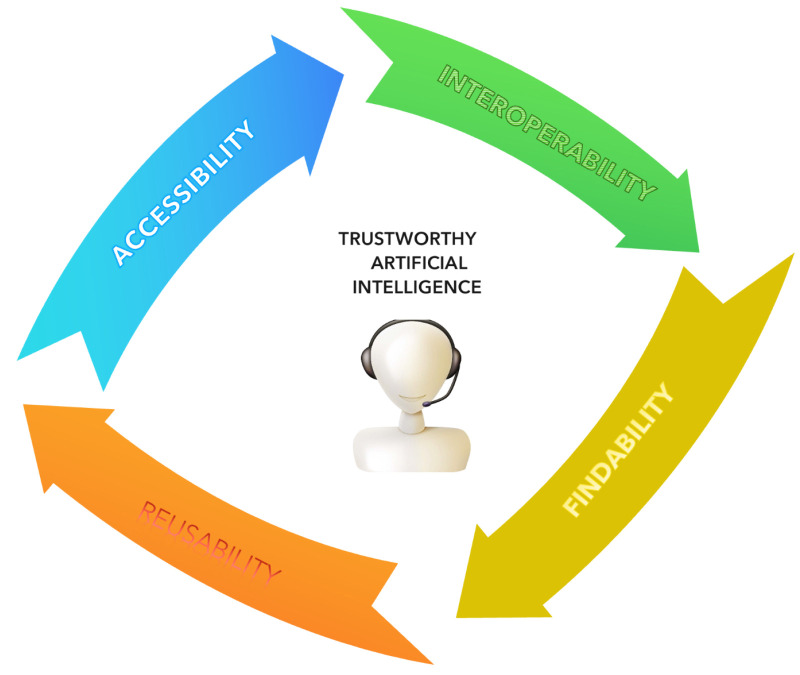
Trustworthy artificial intelligence.

An accurate SaMD application must be standardized: it should produce consistent results when applied to similar datasets, regardless of the user or setting. This adaptability, however, increases the demands for compliance with data protection guidelines and adequate security measures [37]. The EMA (European Medicine Agency), the FDA (U.S. Food and Drug Administration), hospitals, and healthcare providers and manufacturers are all responsible for warranting that SaMD can work across systems. Adherence to the FAIR principles for data management is mandatory: accessibility, interoperability, findability, and reusability [64] (Figure 12).

Specifically for SaMD intended for diagnosis, prevention, monitoring, and treatment, there is a need for clinical and real-world studies; everything must flow for human benefit, with clear and transparent algorithms—the way they reach decisions must be readily understood (explainability and transparency) [31,33,62]. The ‘black box’ is a major challenge in AI, especially with DL algorithms: to achieve trust and clinical adoption, developing AI systems with interpretable and understandable outputs is crucial to integration in clinical settings [13,19,31,33]. Concerns about reliability or a preference for established practices also make healthcare professionals hesitant in AI implementation.

AI systems may inadvertently perpetuate existing biases present in the training data, leading to disparities in care and compromising equitable healthcare delivery [8,31,33,116]. If minority populations are underrepresented in medical datasets, AI tools may be less accurate for these groups, exacerbating health inequities. Bias can be introduced into the clinical decision-making process during training or through decisions made during SaMD design [61]. Addressing this requires deliberate efforts to collect diverse and representative data and implement strategies that mitigate bias in AI development [12,31,33,63,69,89].

Determining responsibility for AI-driven decisions, particularly in surgical contexts, raises ethic–legal questions. Clear guidelines and informed consent when AI tools are involved in patient care are essential [33]. It is important to note that AI may misclassify nodules, leading to unnecessary biopsies or missed cancers [2,17,33]. Responsibility for AI-driven decisions (developers, healthcare providers, and institutions) becomes complex and involves multiple factors and stakeholders, especially when errors occur. Liability depends on the nature of the AI system, its integration into clinical practice, and the specific circumstances of the case. The clinician retains primary responsibility for patient outcomes when assistive-AI provides recommendations, because they ultimately make the final decisions. However, autonomous AI operates with minimal human intervention. When harm results from its use, liability may shift towards the developers or manufacturers, if the system was used as intended and adhered to regulatory standards. Healthcare institutions are responsible for properly integrating AI systems into their workflows, including adequate staff training, regular maintenance, and timely updates. Failure to do so could result in institutional liability. Manufacturers may be liable under product liability laws, if the AI system is found to be defective in design, manufacturing, or lacks proper instructions and warnings. The legal landscape for AI in healthcare is still developing.

Advanced AI-driven robotic surgery systems are not widely available: implementation can be expensive, limiting access in resource-constrained settings [2,32,33]. Integrating AI tools into clinical workflows and EHRs is crucial and can be technically challenging and resource-intensive. Implementing and maintaining AI systems require substantial financial investment, which may not be feasible for all healthcare institutions [31,37].

While AI holds transformative potential for healthcare, addressing these challenges and ethical considerations is imperative to ensuring that its integration promotes health equity, protects patient rights, and maintains public trust. Collaborative efforts to develop robust, transparent, and ethical AI solutions tailored to the unique demands of TS are a priority for AI to become trustworthy.

### 4.2. Future Directions and Emerging Trends

The future may include the development of AI models with interpretable, clinician-friendly explanations. Future directions should address the need for adequate clinical studies and real-world data to demonstrate the safety and suitability of SaMD. AI will streamline LC screening and pulmonary disease diagnosis with explainable AI in LC-CT analysis, multimodal AI (a combination of MRI, CT, LUS, PET, liquid biopsy, genomics, and clinical data), and real-time AI triage systems (where AI will automatically flag high-risk scans, providing instant radiology reports) [2,37,73,95,96,97,98].

AI-powered robotic systems will allow for delicate dissections (simulating tactile sensation) [45,55,117,118]. The use of CT-based lung segmentation models to predict respiratory complications post-surgery, the monitoring of postoperative recovery through the continuous tracking of several variables, and virtual simulations of post-op recovery for personalized rehabilitation are expected in the near future.

In LC treatment, AI will also be able to combine genomic, proteomic, and radiomic data for ultra-personalized therapy, tracking treatment response dynamically (through liquid biopsy and imaging) and providing assessment of long-term survival and recurrence risks in LC patients [2,111]. In the field of clinical research, AI will be able to accelerate drug discovery for novel LC therapies [31].

In summary, AI is driving transformative changes in TS, from enhancing diagnostic accuracy and surgical precision to personalizing treatment plans and innovating rehabilitation methods. As these technologies continue to evolve, they hold the promise of further improving patient outcomes and the overall quality of care. Table 13 shows examples of emerging technologies being developed as SaMD in TS.

## 5. Conclusions

AI is going to be a disruptive technology in many medical fields, affecting clinical decision making, doctor–patient dynamics, and outcomes. TS is one of those fields; AI will reduce diagnostic errors and enhance imaging and predictive analytics and will be able to improve preoperative, intraoperative, and postoperative outcomes in TS patients.

AI in TS is still in its infancy and has limitations: addressing data bias, ethical concerns, and integration barriers and achieving large-scale validation and regulatory approval before widespread adoption are mandatory. Explainability and clinician–AI collaboration must also be a priority for safe integration. Objectivity, high efficiency, multiplicity, and repeatability, combined with imaging, genomics, pathology, EHRs, and other data streams, will transform AI into a powerful comprehensive diagnosis system.

It is believed that AI may change the current medical model. Future AI innovations will focus on real-time analytics and AI-assisted robotic procedures. The clinical impacts of AI in TS remain unclear and unassessed, so additional research is a priority to determine the advantages and disadvantages in the field.

## Figures and Tables

**Table 1:** Diverse range of AI algorithms and their applications in healthcare, showcasing how different methods are tailored to specific tasks and data types.

Category	Algorithm Type	Common Applications	Notable Examples
	Support Vector Machine (SVM)	Classification tasks, disease diagnosis, image analysis	Cancer classification, early Alzheimer’s detection
	Random Forest	Risk prediction, feature selection, clinical decision support	Cardiovascular disease risk modeling
Supervised Learning	Logistic Regression	Binary classification (e.g., disease presence)	Diabetes outcome prediction deling
	Naïve Bayes	Text classification, clinical document tagging	Medical literature triage
	K-Nearest Neighbors (KNN)	Pattern recognition	Patient similarity-based diagnostics
	Decision Trees	Clinical decision-making	Rule-based triage systems
Unsupervised Learning	K-Means Clustering	Patient stratification	Identifying phenotypic clusters in sepsis
	Hierarchical Clustering	Genetic data classification	Genomic sequence analysis
	Principal Component Analysis (PCA)	Dimensionality reduction	Omics data preprocessing
	Q-Learning	Personalized treatment, adaptive trials	Optimizing chemotherapy dosing
Reinforcement Learning	Deep Q-Networks (DQN)	Robotic control, real-time surgical assistance	Autonomous suturing, tool trajectory optimization in robotic-assisted surgery
	Policy Gradient Methods (e.g., PPO, A3C)	Fine-grained motion control, dexterous manipulation in surgery	Enhancing robotic precision and safety during minimally invasive procedures, real-time feedback control
	Model-Based RL	Adaptive strategy learning in robotic systems	Robotic systems adjusting to changing environments, patient-specific anatomy, or tissue feedback during procedures
Deep Learning	Artificial Neural Networks (ANNs)	Complex pattern recognition	ECG interpretation, pathology image classification
	Convolutional Neural Networks (CNNs)	Medical image classification	Radiology: tumor detection, fracture classification
	Recurrent Neural Networks (RNNs)	Time-series data (e.g., vital signs)	ICU monitoring systems, disease progression modeling
Ensemble Methods	Boosting (e.g., XGBoost)	High-performance classification and regression	Heart failure prediction models
	Bagging (e.g., Random Forest)	Reducing variance and enhancing accuracy	Cancer prognosis modeling
	Stacking	Combining multiple models for improved performance	Multimodal diagnostic platforms
	Named Entity Recognition (NER)	Extracting clinical terms, symptoms, and drug names from text	Identifying conditions and treatments from EHRs
	Bag-of-Words/TF-IDF	Feature extraction from medical notes	Creating input vectors for classifiers in radiology reports
Natural Language Processing (NLP)	Word Embeddings (Word2Vec, GloVe, BioBERT)	Semantic understanding, clinical term relationships	Mapping patient descriptions to ICD codes
	Transformer Models (BERT, GPT, BioBERT)	Text summarization, question answering, clinical trial matching	Biomedical Q&A systems, literature summarization, patient eligibility screening
	Topic Modeling (LDA, NMF)	Discovering latent themes in patient notes or research abstracts	Mining medical literature to detect emerging disease trends

**Table 2:** Comparison table with the difference between AI algorithms and Software as a Medical Device (SaMD) in the healthcare context.

Aspect	AI Algorithms	Software as a Medical Device (SaMD)
Definition	Computational models designed to learn patterns from data and make predictions.	Standalone software intended for medical purposes without needing hardware to achieve its function.
Purpose	To perform specific tasks like classification, prediction, pattern recognition.	To diagnose, monitor, prevent, or treat a disease or condition.
Use in Healthcare	Detect tumors, analyze ECGs, predict complications, extract info from clinical notes.	Provide clinical decisions, risk assessments, or alerts to healthcare providers or patients.
Standalone or Not?	Not typically standalone—it’s a component in a larger system.	Standalone software, even if deployed on mobile apps, cloud platforms, or hospital systems.
Requires Regulation?	Not directly regulated unless integrated into a medical device or SaMD.	Yes, strictly regulated by authorities like FDA, EMA, or ANVISA.
Examples	CNNs, RNNs, SVMs, decision trees, BERT-based NLP models.	AI-powered ECG interpretation app, diabetes risk predictor, digital pathology tools.
Medical Claims	Cannot independently make medical claims.	Can make regulated medical claims (e.g., “detects AFib from smartwatch data”).
Clinical Validation	Requires technical validation (accuracy, precision, recall, etc.).	Requires clinical validation (safety, effectiveness, benefit-risk profile).
Lifecycle Oversight	Focus on development, testing, retraining.	Requires full lifecycle management (design, development, deployment, updates, post-market monitoring).
Can It Use AI?	Is AI.	May or may not use AI—can also be rule-based or statistical.

**Table 3:** Common AI-related terms in medical imaging and healthcare: definition, purpose, and examples of use.

Term	Definition	Purpose/Use Case	Example
AI-Based Tools	General term for digital systems that incorporate AI algorithms to support or perform healthcare-related tasks.	Diagnostic support, image analysis, workflow automation.	AI triage tools in radiology that prioritize abnormal chest X-rays.
AI-Driven Radiomics	Radiomics that use AI (especially ML/DL) to automatically extract and interpret quantitative features from medical images.	Predict disease outcome, phenotype tumors, guide personalized treatment.	Predicting lung cancer survival based on CT features using ML.
Radiomics	Process of extracting a large number of quantitative features from medical images using data-characterization algorithms (not always AI-based).	Feature extraction for risk stratification, treatment response prediction.	Texture analysis in MRI to differentiate benign from malignant lesions.
Radiogenomics	Integrates imaging features (radiomics) with genetic or molecular data to identify associations between imaging phenotypes and genomics.	Discover imaging biomarkers that reflect gene expression, guide targeted therapy.	Linking imaging patterns on CT with EGFR mutations in lung cancer.
AI-Augmented Imaging	Imaging processes enhanced with AI for real-time analysis, annotation, or image quality improvement.	Improve speed, accuracy, and confidence of radiologists during interpretation.	Real-time AI overlay for polyp detection during colonoscopy.
Computer-Aided Detection (CAD)	Systems designed to assist in the detection of abnormalities by highlighting suspicious areas in medical images.	Assist radiologists by flagging potential pathology.	CAD for mammography to detect breast cancer.
Explainable AI (XAI)	Methods and techniques to make the decision-making process of AI models understandable and transparent to humans.	Build trust, ensure accountability in clinical AI systems.	Heatmaps (e.g., Grad-CAM) showing regions influencing AI image classification.

**Table 6:** AI-based clinical decision support (CDS) tools combine clinical data with AI algorithms to assist healthcare professionals in making informed decisions about patient care. This table explains what AI-based CDS tools consist of.

	AI-Based Clinical Decision Support Tools
**Definition**	Software tools that use AI (often ML or NLP) to provide evidence-based clinical recommendations.
**Function**	Support clinicians in diagnosis, prognosis, treatment selection, and risk prediction.
**Inputs**	Electronic Health Records (EHR), imaging data, lab results, genomics, clinical notes.
**Core AI Techniques**	ML, DL, NLP, Expert Systems.
**Types**	- Diagnostic support - Prognostic modeling - Treatment recommendation - Alerts & reminders
**Examples**	- AI predicting sepsis risk in ICU - Suggesting personalized chemotherapy regimens - Flagging drug interactions
**Benefits**	- Reduces errors - Enhances decision-making - Supports evidence-based care - Increases efficiency
**Challenges**	- Bias in training data - Explainability of models - Integration with clinical workflows - Regulatory compliance
**Regulation**	Often classified as SaMD and must meet standards set by bodies like FDA or EMA.

AI—artificial intelligence; DL—deep learning; EHR—electronic health records; ICU—intensive unit care; ML—machine learning; NLP—natural language processing.

**Table 13:** Examples of emerging technologies currently being developed as SaMD in thoracic surgery healthcare: AI-based SaMD challenges and potential applications.

Areas of Thoracic Surgery Application	Challenges	AI-Based SaMD and Potential Applications
AI-Based Radiological Imaging Analysis	Regulatory approval (FDA, EMA) due to accuracy and safety concerns. Data bias in AI training, leading to potential misdiagnoses in underrepresented populations. Integration with existing PACS	Assist in detecting and classifying lung diseases (e.g., lung cancer, pneumonia) using CT scans and X-rays. Automated nodule detection and malignancy risk assessment. AI-enhanced image segmentation for preoperative planning in Thoracic Surgery.
AI-Guided Bronchoscopy Navigation	Real-time processing and latency issues in high-resolution imaging. Validation of AI recommendations in clinical practice. Surgeon acceptance and trust in AI-assisted procedures.	AI-assisted SaMD can enhance real-time navigation during bronchoscopies, helping in biopsy guidance and tumor localization. Virtual bronchoscopy software with AI predictive modeling.
AI-Powered Pulmonary Function Testing (PFT) and Spirometry Analysis	Standardization of AI interpretations across different spirometry devices. Ensuring AI-based diagnostics match or exceed pulmonologists’ expertise. Regulatory hurdles for direct patient-facing applications.	AI-driven SaMD for automated interpretation of spirometry and lung function tests. Predictive analytics for early detection of chronic lung diseases like COPD and asthma. Personalized treatment recommendations based on lung function trends.
AI-Enabled Remote Monitoring for Respiratory Patients	Data privacy concerns and compliance with HIPAA/GDPR. Reliability of AI predictions in real-world settings. Interoperability with different wearable devices and EHRs.	AI-powered SaMDs analyze data from wearable devices (e.g., smart inhalers, pulse oximeters) to monitor respiratory conditions remotely. Early detection of exacerbations in COPD and asthma using ML algorithms.
AI for Post-Thoracic Surgery Monitoring and Predictive Analytics	Need for extensive validation before clinical adoption. Potential over-reliance on AI, reducing clinician oversight. Ethical concerns regarding algorithmic decision-making in high-risk patients.	AI-driven risk prediction models for post-surgical complications (e.g., pneumonia, ARDS). Automated detection of post-operative complications using continuous patient monitoring. AI-powered rehabilitation tracking for lung transplant and thoracic surgery patients.

AI—artificial intelligence; ARDS—acute respiratory distress; COPD—chronic obstructive pulmonary disease; CT—computed tomography; EHR—electronic health records; EMA—European Medicine Agency; FDA—federal drug administration; HIPAA/GDPR—health insurance portability and accountability—general data protection regulation; PACS—Picture Archiving and Communication Systems.

## Data Availability

No new data were created or analyzed in this study.
